# Subsurface Microbial Ecology at Sediment-Groundwater Interface in Sulfate-Rich Playa; White Sands National Monument, New Mexico

**DOI:** 10.3389/fmicb.2019.02595

**Published:** 2019-11-12

**Authors:** Mihaela Glamoclija, Steven Ramirez, Kosala Sirisena, Inoka Widanagamage

**Affiliations:** ^1^Department of Earth and Environmental Sciences, Rutgers University, Newark, NJ, United States; ^2^Geophysical Laboratory, Carnegie Institution of Washington, Washington, DC, United States; ^3^Department of Environmental Technology, Faculty of Technology, University of Colombo, Colombo, Sri Lanka; ^4^Department of Geology and Geological Engineering, The University of Mississippi, Oxford, MS, United States

**Keywords:** microbial ecology, playa, subsurface, groundwater, sulfates

## Abstract

The hypersaline sediment and groundwater of playa lake, Lake Lucero, at the White Sands National Monument in New Mexico were examined for microbial community composition, geochemical gradients, and mineralogy during the dry season along a meter and a half depth profile of the sediment *vs.* the groundwater interface. Lake Lucero is a highly dynamic environment, strongly characterized by the capillary action of the groundwater, the extreme seasonality of the climate, and the hypersalinity. Sediments are predominantly composed of gypsum with minor quartz, thenardite, halite, quartz, epsomite, celestine, and clays. Geochemical analysis has revealed the predominance of nitrates over ammonium in all of the analyzed samples, indicating oxygenated conditions throughout the sediment column and in groundwater. Conversely, the microbial communities are primarily aerobic, gram-negative, and are largely characterized by their survival adaptations. Halophiles and oligotrophs are ubiquitous for all the samples. The very diverse communities contain methanogens, phototrophs, heterotrophs, saprophytes, ammonia-oxidizers, sulfur-oxidizers, sulfate-reducers, iron-reducers, and nitrifiers. The microbial diversity varied significantly between groundwater and sediment samples as their temperature adaptation inferences that revealed potential psychrophiles inhabiting the groundwater and thermophiles and mesophiles being present in the sediment. The dynamism of this environment manifests in the relatively even character of the sediment hosted microbial communities, where significant taxonomic distinctions were observed. Therefore, sediment and groundwater substrates are considered as separate ecological entities. We hope that the variety of the discussed playa environments and the microorganisms may be considered a useful terrestrial analog providing valuable information to aid future astrobiological explorations.

## Introduction

Hypersaline environments harbor very diverse ecosystems that may range from soda lakes, saltpans, salars, hypersaline springs, playas, and ancient salt deposits (Friedman and Krumbein, 1985; Reynolds et al., 2007; Oren et al., 2009). Consequently, the ecology of hypersaline environments has been extensively investigated, especially the surface water column of playas, and the sediments after the wet seasons, which is the period when organisms flourish (Sorenson et al., 2004, 2005; Mesbah et al., 2007; Costa et al., 2008; Navarro et al., 2009; Oren et al., 2009; Makhdoumi-Kakhki et al., 2011). Many among the studies had focused on different ecological and chemical aspects of the stratification of microbial mats living in wet hypersaline sediments (Sorenson et al., 2004, 2005; Oren et al., 2009; Vogel et al., 2009) while sediments and/or groundwater were subject of the fewer investigations (Schulze-Makuch, 2002; Navarro et al., 2009; Pen-Mouratov et al., 2011; Sirisena et al., 2018). Therefore, to fully understand the microbial ecology of playa setting it is crucial to explore the microbial communities living in different substrates of playa (i.e., sediments and groundwater) during the drought period, characterized by these particularly harsh weather conditions.

Playas are intracontinental basins in which drought periods exceed wet periods that are characterized by precipitation and water inflow (Cooke et al., 1993). Due to the ephemeral nature of these environments, the microbial population is composed of organisms that can survive drought, as well as, temporary freshwater to saline and hypersaline conditions that alternate throughout the year (Ventosa et al., 2008). Previous studies have revealed diverse microbial communities living at similar saline environments with the phyla *Bacteroidetes*, *Firmicutes*, *Actinobacteria*, *Proteobacteria*, and *Euryarchaeota* generally being the most common (Mesbah et al., 2007; Costa et al., 2008; Navarro et al., 2009; Makhdoumi-Kakhki et al., 2011; Babavalian et al., 2013). Furthermore, halophilic microbes have been found as particularly abundant (Babavalian et al., 2013). The objective of this study is to investigate the composition of microbial communities living in the playa ecosystem at the WSNM during the dry season in exclusively hypersaline settings along the steep subsurface environmental gradients.

**FIGURE 1:**
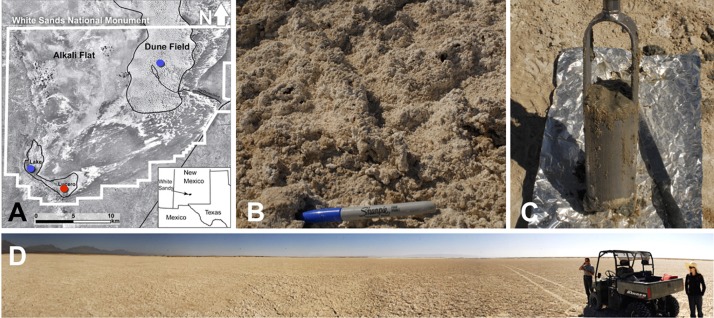
**(A)** A map showing the WSNM area and the sampling points. The red dot denotes the location where the sediment (1–14) and one groundwater sample (GW-1) were collected. The blue dots indicate the location of groundwater samples in Lake Lucero (bottom blue dot, GW-2) and the dune field (upper blue dot, GW-3 and GW-4). **(B)** The surface salt crust at the sampling area. **(C)** A sample of sediment groundwater interface sampled at about 1 m depth. **(D)** The sampling location at the central area of Lake Lucero playa.

The study area is the White Sands National Monument (WSNM) in New Mexico (U.S.), the site that contains the world’s largest gypsum dune field. To the west of the dunes, stretches the Alkali Flat. That is a large, flat, and mostly unvegetated space that hosts about 20 playas, including Lake Lucero (Figure 1). Lake Lucero is the largest among the playas, and it occupies the southern part of the Monument (Langford, 2003; Kocurek et al., 2007). Since Lake Lucero is the lowest topographic point at WSNM, evaporites accumulate here and build thick deposits that create a hypersaline environment (Kocurek et al., 2007). Previous studies, including the analysis of the nearby WSNM dune deposits, have indicated the presence of *Cyanobacteria* as primary producers and as a diverse microbial community capable of cycling nitrogen and sulfur compounds (Glamoclija et al., 2012). Only a few studies have examined the microbial ecology of Lake Lucero’s sediments and groundwater specifically (e.g., Schulze-Makuch, 2002; Sirisena et al., 2018). Lake Lucero is a wet playa with the groundwater table relatively close to the surface; during the dry season, surface moisture is provided by capillary action (Cooke et al., 1993; Reynolds et al., 2007; Szynkiewicz et al., 2010; Newton and Allen, 2014). This process provides much-needed water to microbial communities on the playa surface, as well as a geochemically active environment on the surface and subsurface that organisms may take advantage of Cooke et al. (1993), Glamoclija et al. (2012). Lake Lucero’s groundwater is influenced by a regional groundwater system more so than the rest of the WSNM, which further contributes to the salinity (Newton and Allen, 2014). The seasonal variations in water availability, wind erosion, and the hypersalinity pose potential challenges for life in this environment (Anton et al., 2008; Newton and Allen, 2014).

Desert environments and geomorphology of the WSNM have been discussed as terrestrial analog to Martian sedimentary sequences (Grotzinger et al., 2005; Szynkiewicz et al., 2010; Glamoclija et al., 2012). Sedimentary beds produced by past playa settings have been inferred to exist on Mars, and considering Martian geological history the historic increase in desertification and presence of evaporitic processes may mark some of the last habitats on the red planet (e.g., Andrews Hanna et al., 2010). In the light of Mars 2020 mission flying to past fresh water lake that may hold lacustrine and post-lacustrine lithologies, it is crucial for us to understand where life proliferates in ephemeral lake settings, such as this of Lake Lucero, and which strata may or may not hold the evidence of extinct or present life.

This study aims to evaluate variations in the microbial ecology along the 1.25 m depth profile, geochemical gradients, changes in mineralogy, and substrate (sediment vs. groundwater). Our sampling provides a snapshot of ecology during the dry season, and the sampled depth profile reached groundwater table that coincides with the hard crust of coarse gypsum that we could not sample using auger drilling method. We intend to answer how environmental parameters such as the presence of shallow groundwater table, solar radiation, and geochemistry may influence the distribution of the organisms and to inquire as to which settings are essential and will condition the community structure in these sediments. Furthermore, we have investigated whether groundwater and sediments represent separate ecological entities.

## Materials and Methods

### Sampling Procedures

**FIGURE 2:**
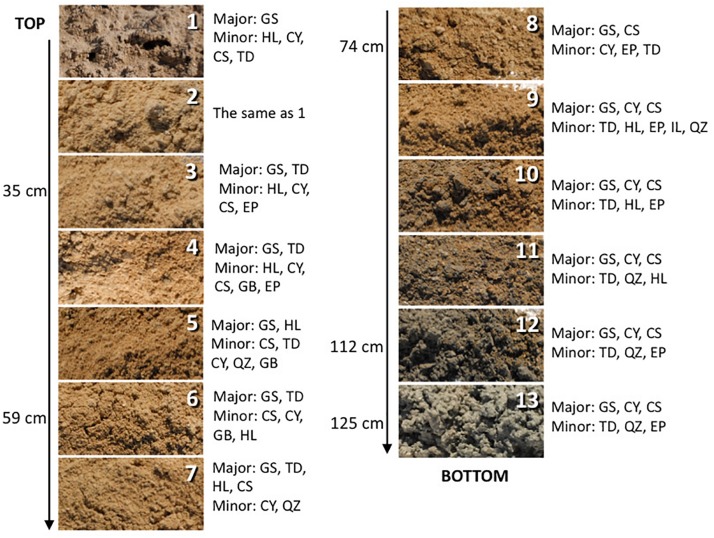
The depth profile displaying changes in sediment appearance (texture and color) as the depth increases. Next to the photo of the sample are listed major and minor mineral compositions. Sample 14 is not pictured and shown here as it is mineralogically the same as sample 13 but it was more liquid due to higher mixture with groundwater. The letter symbols for minerals are: GS, gypsum; HL, halite; CY, clay; CS, celestine; TD, thenardite; EP, epsomite; GB, glauberite; QZ, quartz.

Sediment and groundwater samples were collected in March 2013 from three locations at the WSNM (Figure 1). The sediment sampling strategy was designed to assess the depth profile of playa deposits to capture different evaporation lithologies formed at Lake Lucero. The sampling location (N 32° 41.111′; W 106° 24.093′ ± 3 m) is an approximate topographic low within Lake Lucero where the lake surface water has had the opportunity to last longer time than in other areas of the playa, and the microbial communities had the most opportunity to colonize and diversify within the evaporitic sediments. Our initial attempt to manually drill had failed due to sediment characteristics (too hard and sticky). Instead, we sampled a 125 cm deep lithological profile using an auger device. The auger device was pre-cleaned to prevent contamination (Eigenbrode et al., 2009). Within this profile, we had subsampled 14 lithologically different samples (Figure 2), which were divided by depth as consistently as possible and then placed in Falcon tubes and sterile plastic bags. We discovered that the water table was located at 125 cm depth (Figures 1, 2). The coarse gypsum at the bottom of the hole was too hard to auger through, and our sampling ended at this level. The groundwater within the drilled hole was left to settle until the next day and collected into a pre-cleaned, 4 L carboy using a manual vacuum pump (GW-1). Three other groundwater samples were collected from previously installed piezometers: one from a southernmost location in Lake Lucero (N 32° 42.167′; W 106° 26.960′ ± 3 m) (GW-2), and two from the dune field (N 32° 49.721′; W 106° 15.972′ ± 3 m). Dune field piezometers were installed for monitoring of shallow and deep aquifers, as clarified by WSNM park management. The shallow aquifer sample primarily contained meteoric water (GW-4), whereas the deep aquifer sample was the brines (GW-3) (see Table 1). The groundwater samples were filtered (4 L for each sample) using 0.22 μ membrane filters within a few hours of the collection. Filters were placed in sterile tubes, and all samples were held at 4°C in a refrigerator during the fieldwork and the transportation back to the laboratory, where they were stored in the freezer at −20 and −80°C until further processing.

**TABLE 1:** Groundwater field measurements.

**Sample location**	**Lake Lucero – central (GW-1)**	**Lake Lucero – south (GW-2)**	**Dune field – deep aquifer (GW-3)**	**Dune field – shallow aquifer (GW-4)**
Temperature (°C)	17.7	15.0	17.5–18.5	16.8–18.2
pH	7.09	7.84	7.02	7.40
Conductivity (mS)	59.0	139.9	37	11.98

### Mineral Assemblages

Main mineral phases were identified by X-ray diffraction of powdered dry and dump wet samples using a Bruker D8 Advance Eco, equipped with a Cu-Kα radiation source and a LynxEye XE detector. Samples were afterward analyzed using EVA software. Scanning Electron Microscope (SEM) with Energy Dispersive X-ray Spectroscopy (EDS) Hitachi S-4800 was used to search for the presence of microbial morphologies or biofilm and to analyze their elemental composition and minor mineral phases and precipitates. All samples were subsampled three times for the SEM-EDS analysis. Once dried, the samples were coated with Iridium and analyzed using 25.0 and 15.0 kV voltage, 20 μA under standard vacuum, and working distance ranged from 9 to 13 mm.

### Geochemistry

All of the collected samples were analyzed for Mg, Sr, Fe, Na, K, and Ti concentrations using ICP-OES. One gram (dry weight) of the sample was mixed with repeated additions of nitric acid (20%) up to 10 ml (following acid digestion of soils) for 4 days with periodic sample shaking and heating. Samples were filtered, and the filtrates were diluted with deionized water and volumes brought up to 30 ml and adjusted total acid to 3–5% (v/v) for ICP-OES analyses. The analytical reproducibility was calculated as standard deviation and was within a range of 0.1459 to 8.0000 for more abundant cation concentrations, on average 3.53 for all the analyzed samples.

The ammonium (${\text{NH}}_{4}^{+}$) and nitrate + nitrite (${\text{NO}}_{3}^{-}$ + ${\text{NO}}_{2}^{-}$) concentrations from the deposits were assessed using colorimetric methods. One gram of sample was mixed with 10 ml of 2N potassium chloride (KCl) and left in the solution for 24 h at room temperature while shaking periodically. The supernatant was decanted into clean Falcon tubes. A range of 0, 5, 10, 25, 50, and 100 μM solutions were prepared for ammonium sulfate [(${\text{NH}}_{4}^{+}$)_2_SO_4_] and sodium nitrate (NaNO_3_) solutions to be used as standards. The absorbance of each sample was measured at least in triplicates using a GENESYS 10Bio spectrophotometer; 640 nm was used for ammonium and 540 nm for nitrate. The ${\text{NH}}_{4}^{+}$ concentration of the extracts was determined by the alkaline hypochlorite/phenol nitroprusside method, after the addition of sodium citrate to prevent the precipitation of calcium and magnesium salts (Solorzano, 1969). The analytical reproducibility for ${\text{NH}}_{4}^{+}$ measurements was calculated as standard deviation and was within a range of 0.001 to 0.033, on average 0.014 for all the analyzed samples. The ${\text{NO}}_{3}^{-}$ + ${\text{NO}}_{2}^{-}$ concentrations were measured using the Nitrate Test kit (LaMotte, MD, United States) according to the manufacturer’s instructions. This method does not allow for separate ${\text{NO}}_{2}^{-}$ detection, and therefore the results are reported as a sum of ${\text{NO}}_{3}^{-}$ and minor ${\text{NO}}_{2}^{-}$, which will henceforth be referred to as Nitrates ${\text{NO}}_{3}^{-}$. The analytical reproducibility for ${\text{NO}}_{3}^{-}$ measurements was calculated as standard deviation and was within a range of 0.032 to 0.147, on average 0.076 for all the analyzed samples.

### Nucleic Acid Extraction and Polymerase Chain Reaction

Sediment DNA extractions were carried out from approximately 0.5 g of powdered sediment sample, using Qiagen DNeasy^®^ PowerSoil DNA Isolation Kit. Modifications to the manufacturer’s protocol were made as described in Sirisena et al. (2018) to improve the extraction efficiency. The powdered sample was incubated at 70°C for 30 min in a Bead Tube that contained a bead solution from the kit. The incubated mixture was vortexed for 5 min and centrifuged for 30 s at 10,000 × *g*. This modification helps to mechanically separate microbial cells from the mineral substrate and eliminate most of the inhibitors to the DNA extraction (clays, gypsum, halite, and other salts) (Glamoclija et al., 2012). The supernatant was used as the starting material for the DNA extraction that was carried out according to the manufacturer’s instructions. DNA extractions from all the samples were conducted at least in triplicates to account for sample heterogeneity. Negative (no sample) and positive (garden topsoil) extraction controls were used to ensure the extraction quality. Filters containing particles from groundwater samples were cut in small pieces using a sterile knife. The pieces were placed in the bead-beating tube using sterile tweezers and processed in the same way as other samples. The recovered DNA was stored at −20°C until further processing.

Microbial genomic DNA extraction from all three domains (archaea, eubacteria, and eukayota) was verified by polymerase chain reaction (PCR) using domain-specific 16S- and 18S- rRNA gene primers (Eubacterial B27-F and 1429-R (DeLong, 1992; Madden et al., 2007), Archaeal 8A-F and 1513U-R (Eder et al., 1999; Huber et al., 2002), and Eukaryal Euk1-F and Euk-R2 (Potvin and Lovejoy, 2008).

### Illumina MiSeq 16S and 18S Amplicon Sequencing

A pair of universal primers: 515F (5′ GTG CCA GCM GCC GCG GTA A 3′) and 806R (5′ GGA CTA CHV GGG TWT CTA AT 3′) (Caporaso et al., 2012; Itoh et al., 2014; Pylro et al., 2014; Wu et al., 2015) was used to sequence the variable region V4 of the prokaryotic 16S rRNA gene for the detection of bacteria and archaea. Similarly, an another pair of universal primers: Euk7F (5′ AAC CTG GTT GAT CCT GCC AGT 3′) (Medlin et al., 1988; Auld et al., 2016) and Euk570R (5′ GCT ATT GGA GCT GGA ATT AC 3′) (Weekers et al., 1994; Auld et al., 2016) was used to sequence the variable region V1–V3 of the eukaryotic 18S rRNA gene to explore the eukaryotic microbial communities in the samples. Initial PCR amplification was conducted using approximately 15 ng of microbial genomic DNA from each sample using a forward primer with a unique barcode. PCR conditions comprised of an initial denaturation at 94°C for 3 min, 30 cycles of denaturation at 94°C for 30 s, annealing at 53°C for 40 s and elongation at 72°C for 1 min each, followed by a final elongation at 72°C for 5 min. After amplification, PCR products were checked in 2% agarose gel to determine the success of amplification. Then the PCR products with unique barcode for each sample were pooled together in equal proportions based on their molecular weight and DNA concentrations. Pooled samples were purified using calibrated Agencourt AMPure XP beads. This pooled and purified PCR product was used to prepare Illumina MiSeq DNA library according to the manufacturer’s guidelines. The targeted variable regions of the 16S and 18S genes were sequenced on Illumina MiSeq platform (Caporaso et al., 2012; Garcia-Mazcorro et al., 2016).

### Bioinformatics and Statistical Analysis

The two FASTQ files (R1 and R2) resulted from paired-end sequencing contained forward and reverse reads, respectively. The quality filtering and downstream sequence analysis were performed using Mothur v1.37.2 program (Schloss et al., 2009), according to the Standard Operating Procedure (SOP) outlined on https://www.mothur.org/wiki/MiSeq_SOP and customized as described by Sirisena et al. (2018). Briefly, the forward and reverse paired-end reads in R1 and R2 FASTQ files were merged to create consensus reads (contigs).

The following quality parameters were applied to eliminate low quality reads: (1) reads of a total quality score less than 25 were discarded; (2) reads with more than 2 bp mismatches in primers and more than one base pair mismatch in barcodes were eliminated. The barcodes and forward and reverse sequencing primers were trimmed from the contigs. The unique sequences were aligned to the SILVA reference alignment (release 123) (Quast et al., 2013). The detection and removal of Chimeric sequences were done using the UCHIME program within Mothur (Edgar et al., 2011). The unique sequences were classified to taxonomic levels using SILVA reference database (release 123) (Quast et al., 2013) with a cut-off of 80% of the bootstrap value. This enabled us to remove sequences related to “Chloroplast, Mitochondria, Eukaryota and Unknown” taxonomic lineages from bacteria and archaea analysis, and “Chloroplast, Mitochondria, Bacteria, Archaea and Unknown” lineages from for eukaryotic analysis. Subsequently, quality-filtered bacterial and archaeal unique sequences were clustered into operational taxonomic units (OTUs) at 97% similarity cut-off level using the average neighbor algorithm. For eukaryotic analysis, the quality-filtered unique sequences were clustered into “phylotypes” based on their taxonomy as opposed to the percentage similarity among sequences used in the OTU approach. Then, the abundances of OTUs/phylotypes in each sample were computed with the taxonomic identity up to the genus level for each OTU. The OTUs and phylotypes that were not classified up to lower taxonomic ranks (i.e., genus level) were further identified by manual BLASTn search in the NCBI Genomic Survey Sequences database. The singleton OTUs and phylotypes were not considered for subsequent diversity analyses.

The number of OTUs, the diversity indices: Shannon and Simpson; evenness indices: Shannon and Simpson; estimated richness: Chao 1 and ACE, and Good’s coverage for each sample were calculated separately for bacteria and archaea using Mothur v1.37.2. PRIMER-7 software package was used to analyze changes in microbial community structure with ANOSIM and SIMPER analyses (Clarke and Gorley, 2015). ANOSIM tests the null hypothesis that the average rank similarity between objects within a group and objects from different groups is the same by producing a *p*-value and a test statistic (R) between −1 and 1, where 0 indicates the null hypothesis is true, and 1 shows a high degree of dissimilarity (Rees et al., 2004). SIMPER analysis facilitates the identification of OTUs that are responsible for contributing to community structure difference between individual samples and groups of samples (Rees et al., 2004).

The bacterial and archaeal community composition patterns across depth gradients as well as between sediment and groundwater were investigated by hierarchical cluster analysis using PRIMER-7 package (Clarke and Gorley, 2015). Briefly, the OTU abundances in each sample were standardized to the sum of the sample, and a similarity matrix between samples was constructed based on the Bray–Curtis similarity matric. A hierarchical cluster analysis was performed using the group average linkage method.

### Metabolic Inference From 16S Taxonomic Data

The predicted metabolic functions of bacterial and archaeal communities in samples were determined using METAGENassist web server tool (Arndt et al., 2012). Briefly, the OTUs with same taxonomic assignment were combined. Metabolic inference was conducted considering the taxonomic information available to lowest specified taxonomic rank. The dendrograms and heatmaps were constructed using Spearman distance and Ward linkage algorithm to explore the metabolic profile pattern of the prokaryotic microbial communities in each sample.

## Results

### Mineralogy

The sediments analyzed for this study are predominantly composed of gypsum (CaSO_4_⋅2H_2_O). Additionally, the surface crust contains thenardite (Na_2_SO_4_), halite (NaCl), and a minor amount of clay minerals. Along the profile relatively minor amounts of epsomite (MgSO_4_⋅7H_2_O), glauberite [Na_2_Ca(SO_4_)_2_], celestine (SrSO_4_), and quartz (SiO_2_) are detected too. Below the surface, a light brown mixture of gypsum and clay are identified, at about 60 cm deep the reddish clays, rich in iron oxides, were detected, and at about 1-m deep dark gray clay occurs, and it becomes thick and sticky just above the coarse gypsum strata (Figure 2). The bottom two samples are coarse gypsum and minor dark colored clay. The mineralogical observations reported here are mostly consistent with those previously reported (e.g., Langford, 2003). No obvious microbial morphologies or biofilms were observed in the samples using SEM technique, indicating very low biomass in the analyzed samples. Also, there is a possibility that microorganisms were included in the salt minerals and therefore invisible to SEM technique.

### Geochemistry

**FIGURE 3:**
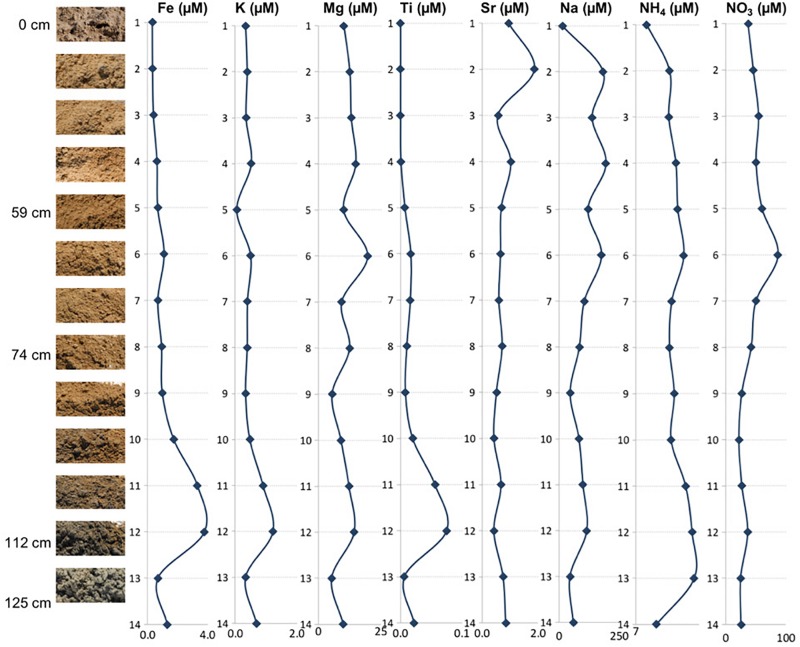
Depth profile illustrating relationships between the mineral assemblages and concentrations of elemental ions, ammonium, and nitrates.

The ${\text{NH}}_{4}^{+}$ and ${\text{NO}}_{3}^{-}$ concentrations revealed that all of the samples had more ${\text{NO}}_{3}^{-}$ than ${\text{NH}}_{4}^{+}$ (Figure 3 and Table 2), indicating the presence of aerobic conditions throughout the depth profile and the potential presence of nitrifying organisms. The ${\text{NO}}_{3}^{-}$ concentrations increased between the surface (23.06 ppm) and 60 cm depth, where it reached its highest value of 53.90 ppm (sample 6) after which the concentrations declined to about 16 ppm. The ${\text{NH}}_{4}^{+}$ levels are much less varying (1.50 to 2.59 ppm), increase with the depth, and it appears that they might be also influenced by lithology too (e.g., ${\text{NO}}_{3}^{-}$ increased in the presence of clays).

**TABLE 2:** Cation concentrations for the soil samples from White Sand Monument.

**Sample**	**Fe (μM)**	**K (μM)**	**Mg (μM)**	**Ti (μM)**	**Sr (μM)**	**Na (μM)**	**${\text{NH}}_{4}^{+}$ (μM)**	**${\text{NO}}_{3}^{-}$ (μM)**
1	0.31	0.34	10.17	BDL	0.95	15.04	8.34	37.19
2	0.33	0.39	12.51	BDL	1.87	182.92	11.26	45.93
3	0.39	0.35	13.08	BDL	0.57	137.94	11.16	54.47
4	0.63	0.52	14.85	BDL	1.03	195.05	12.06	50.53
5	0.68	0.05	10.02	0.01	0.69	120.57	12.31	60.50
6	1.10	0.50	19.58	0.02	0.66	176.42	13.07	86.93
7	0.69	0.40	9.27	0.02	0.60	105.56	11.53	50.46
8	0.93	0.39	12.38	0.01	0.72	85.04	11.25	42.48
9	0.98	0.34	5.42	0.01	0.51	47.33	11.85	26.86
10	1.74	0.47	8.96	0.02	0.43	83.36	11.45	22.05
11	3.32	0.90	12.15	0.06	0.68	99.93	13.31	27.12
12	3.79	1.22	14.18	0.08	0.42	115.25	14.13	36.47
13	0.69	0.34	5.22	0.01	0.75	46.36	14.40	24.94
14	1.31	0.69	9.91	0.02	0.84	59.40	9.58	25.42

*The higher numbered samples are at greater depths (i.e., 1 is from the surface, 14 is from the groundwater). “BDL” stands for Below Detection Limit.*

The concentrations of the examined ions throughout the sediment column (Figure 3 and Table 2) revealed specific patterns. Sodium and magnesium concentrations generally decrease with depth, which directly reflects the variety and contribution of salts other than gypsum to the examined lithologies. Sodium and magnesium derive mostly from halite, thenardite, epsomite, and glauberite. The prevalence of salt is generally consistent with the expectation that evaporitic action would result in higher salinity at the surface (Cooke et al., 1993). Iron concentrations generally increased in deeper sediments, and the same is true for titanium concentrations. The increase of Fe and Ti with depth may be related to the diagenetic processes and the presence of different clays (reddish, dark gray). For example, the change in concentrations of K, Mg, Na, and ${\text{NO}}_{3}^{-}$ within horizons corresponding to samples 6 and 12 are characterized by the presence of sticky clays. Sticky clays result in high concentrations of monovalent cations (e.g., Na), which generate large hydration shells which reduce pore space and thus inhibit microbial transport, water flow, and soil aeration (Maier et al., 2009). Therefore, sticky clays can alter local geochemical conditions and microbial ecology. The K concentrations were relatively low and exhibited a trend similar to that of Fe, Mg, and Ti (Figure 3). The general changes in cation concentrations noted here are consistent with the observations made in SEM/EDS. Strontium in the samples is related to the presence of the mineral celestine (SrSO_4_), the detected concentrations are consistent with SEM/EDS observations as celestine is observed as a minor mineral component in the samples. Strontium concentrations would likely be higher during the wet season due to increased dissolution (Ichikuni and Musha, 1978). Additionally, the strontium component in these samples likely derives from the local groundwater brines that increase the salinity of this playa.

### Taxonomy

**FIGURE 4:**
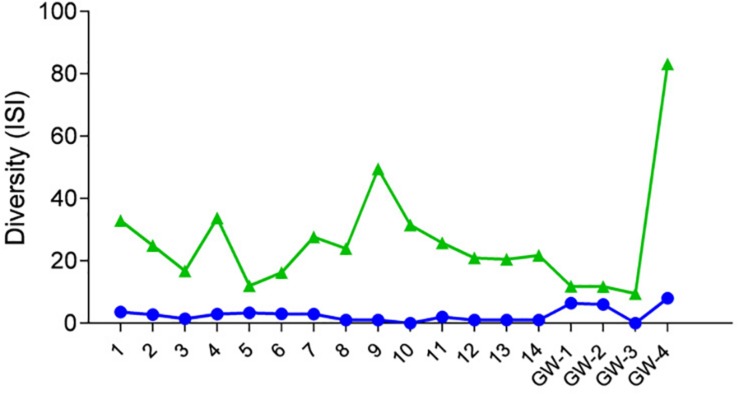
Diversity of samples based on the Inverse Simpson Index (ISI), green triangles represent bacteria, and blue dots represent archaea.

**FIGURE 5:**
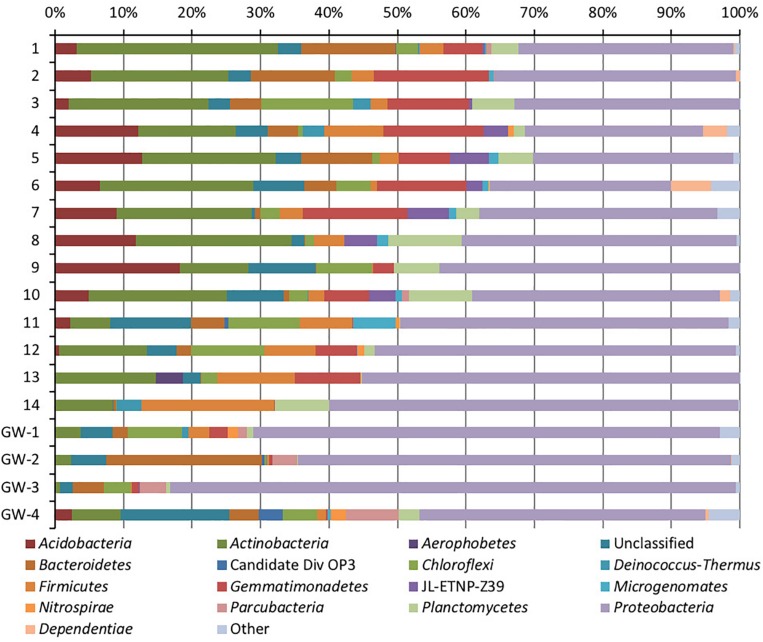
Phylum-level distribution of bacteria in sediments (1 to 14, the numbers increase with the depth) and groundwater (GW-1: on the bottom of the drilled hole; GW-2: piezometer in Lake Lucero and GW-3 and -4: dune deep and shallow groundwater respectively).

After the quality filtration, a total of 7627 OTUs for bacteria, 541 OTUs for archaea, and 34 phylotypes for eukaryota are obtained from the 14 sediment samples and four groundwater samples. Based on the number of OTUs, raw sequences, and diversity analysis, bacteria were the dominant and a diverse domain, especially within the sediment column (Figures 4, 5).

**FIGURE 6:**
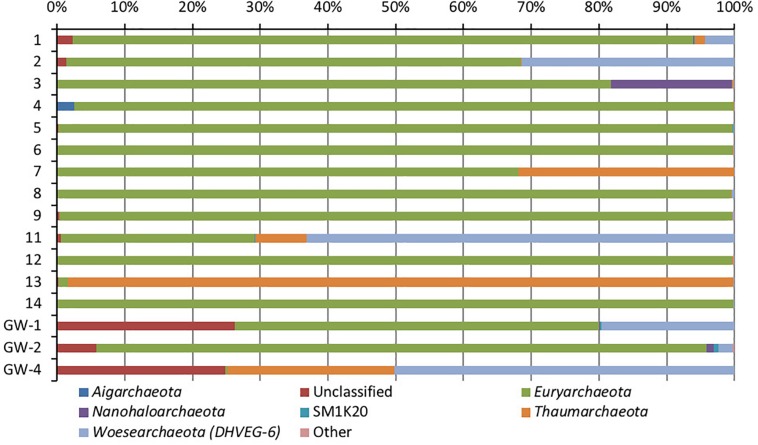
Phylum-level distribution of archaea, in sediments (1 to 14, the numbers increase with the depth) and groundwater (GW-1: on the bottom of the drilled hole; GW-2: piezometer in Lake Lucero and GW-3 and -4: dune deep and shallow groundwater respectively). Samples that contained no archaeal DNA are excluded.

**FIGURE 7:**
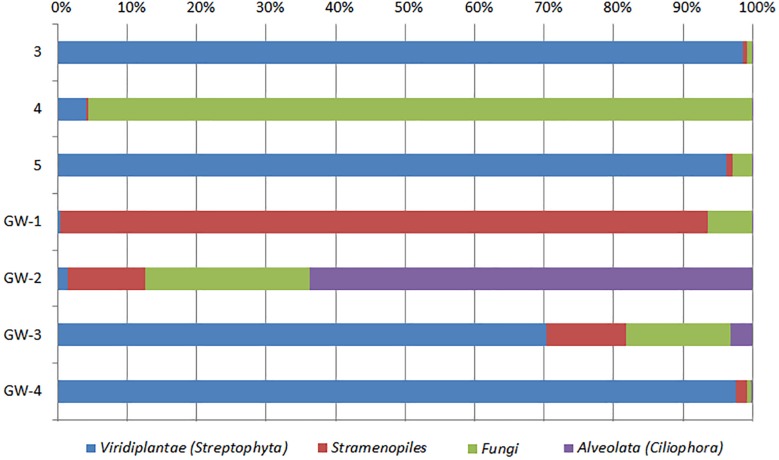
Distribution of eukaryotic groups in sediments (1 to 14, the numbers increase with the depth) and groundwater (GW-1: on the bottom of the drilled hole; GW-2: piezometer in Lake Lucero and GW-3 and -4: dune deep and shallow groundwater respectively). Samples that contained no eukaryotic DNA are excluded.

Based on bacterial taxonomic composition, *Proteobacteria, Acidobacteria, Actinobacteria, Bacteroidetes, Firmicutes*, and *Gemmatimonadetes* are the most dominant phyla (Figure 5). The most dominant *Proteobacteria* accounted for 45% of all bacterial OTUs, and the most dominant within this phylum are *Gammaproteobacteria* (55% of *Proteobacteria* OTUs) and *Alphaproteobacteria* (27%). Archaea are less diverse than the bacteria, and the most dominant archaeal phyla are *Euryarchaeota* and *Thaumarchaeota* (Figure 6). *Euryarchaeota* constitutes approximately 72% of the archaeal OTUs. Eukaryota is identified in three sediment samples and all the groundwater samples, with the most prevalent *Viridiplantae* and *Fungi* (Figure 7). The eukaryotic communities showed very low diversity (Figure 7).

**FIGURE 8:**
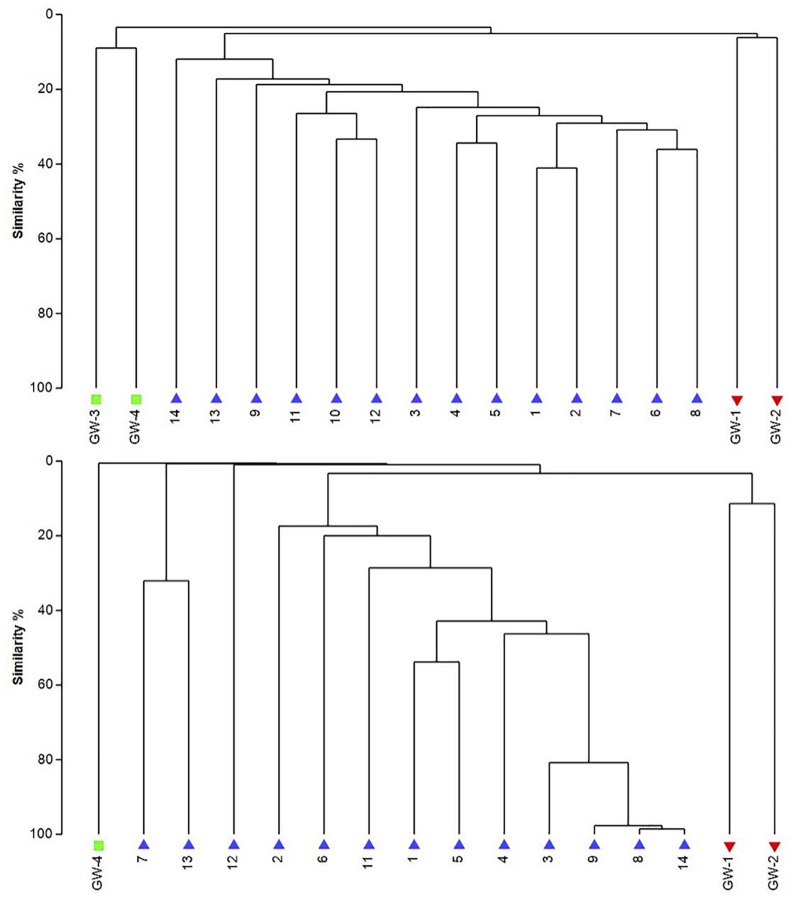
The UPGMA dendrograms of bacteria **(top)** and archaea **(bottom)** based on OTU abundance. The blue triangle is for sediment (1 to 14), the red inverted triangle is for playa groundwater (GW-1, GW-2), and the green square is for dune groundwater (GW-3, GW-4).

The dendrogram was utilized to segregate the samples based on the microbial community structure (OTU abundances). Bacterial clustering revealed that playa groundwater (GW-1 and GW-2) and dune groundwater (GW-3 and GW-4) were different from each other and in general groundwater clustered separately from the sediments (Figure 8). The SIMPER analysis indicates that sample 1 had a higher abundance of OTUs than other sediment samples. A high number of OTUs belongs to genera *Staphylococcus* and *Pseudomonas*. Overall sediment and groundwater were found to be significantly different (SIMPER indicates about 94% the lowest dissimilarity value among any two samples). The ANOSIM analysis confirms the clustering pattern by producing a sample statistic (R) value of 0.99 with a *p*-value < 0.001, which indicates that playa sediments, playa groundwater, and dune groundwater are very different from each based on bacterial community structure. The SIMPER within-group similarities suggest that there is low similarity within the groups; 21.36% for sediments, 8.99% for the dune groundwater (GW-3 and GW-4), and 6.16% for the playa groundwater (GW-1 and GW-2). Based on SIMPER clustering, we can observe that different types of substrates host three differentiated clusters of Bacterial organisms as visible in the dendrogram, Figure 8. Additionally, it appears that samples adjacent to each other (e.g., samples 1 and 2, or samples 4 and 5) are more similar to each other than samples that are physically distant (e.g., samples 1 and 2 are more similar to each other than to sample 14 or sample 13). This style of clustering may indicate that the sediment lithology, geochemistry, or depth may influence the clustering and, therefore, the composition of the organisms within the sediment column. The sediment group was differentiated from the other groups mainly by OTUs classified as *Acidimicrobiales* OM1 clade, *Pseudomonas*, uncultured Sva0071 (*Gammaproteobacteria*), *Delftia* (*Betaproteobacteria*), unclassified *Gammaproteobacteria*, and unclassified *Actinobacteria*. The dune groundwater was differentiated mostly by OTUs classified as *Pseudomonas*, *Sphingobium* (*Alphaproteobacteria*), unclassified *Rhodobacteraceae* (*Alphaproteobacteria*), unclassified JG30-KF-CM66 (*Chloroflexi*), *Seohaeicola* (*Alpharoteobacteria*), and *Methylotenera* (*Betaproteobacteria*). The playa groundwater was differentiated by OTUs classified as *Halomonas* (*Gammaproteobacteria*), *Marinobacter* (*Gammaproteobacteria*), *Thiomicrospira* (*Gammaproteobacteria*), *Sediminimonas* (*Alphaproteobacteria*), uncultured E6AC02 (*Bacteroidetes*), unclassified *Gammaproteobacteria*.

Similar to bacteria, the archaeal communities clustered in three separate groups too. The ANOSIM indicate R of 0.62 with a *p*-value < 0.01, implying that the sample groups are moderately different among each other. However, this result may result from significant separation within the sediment group; since group-to-group comparisons in SIMPER showed that archaeal communities differed greatly between three different habitats just as the bacterial communities do (the lowest dissimilarity value between any two groups is about 97%). Within-group similarities were low: 22.61% for sediments and 11.42% for the playa groundwater. The sediment group was differentiated from the others mainly by OTUs classified as unclassified *Thermoplasmatales* (*Euryarchaeota*), *Marine Group I* (*Thaumarchaeota*), and *Halapricum* (*Euryarchaeota*). The deep dune aquifer sample was differentiated due to OTUs classified as *Marine Group I* (*Thaumarchaeota*), unclassified *Woesarchaeota*, and an unclassified archaean. The playa groundwater was differentiated by OTUs representing unclassified *ST-12K10A* (*Methanomicrobia*), genus *Candidatus Halonobonum* (*Euryarchaeota*), and an unclassified archaean.

The eukaryotes exhibit very limited distribution as they were only observed in three shallow sediment samples and all the groundwater samples. The most prevalent eukaryotic phyla were *Viridiplantae* and *Fungi* (Figure 7). The low quantity of eukaryotic sequences and their absence from many samples makes it unfeasible to examine trends in the community composition in detail.

**FIGURE 9:**
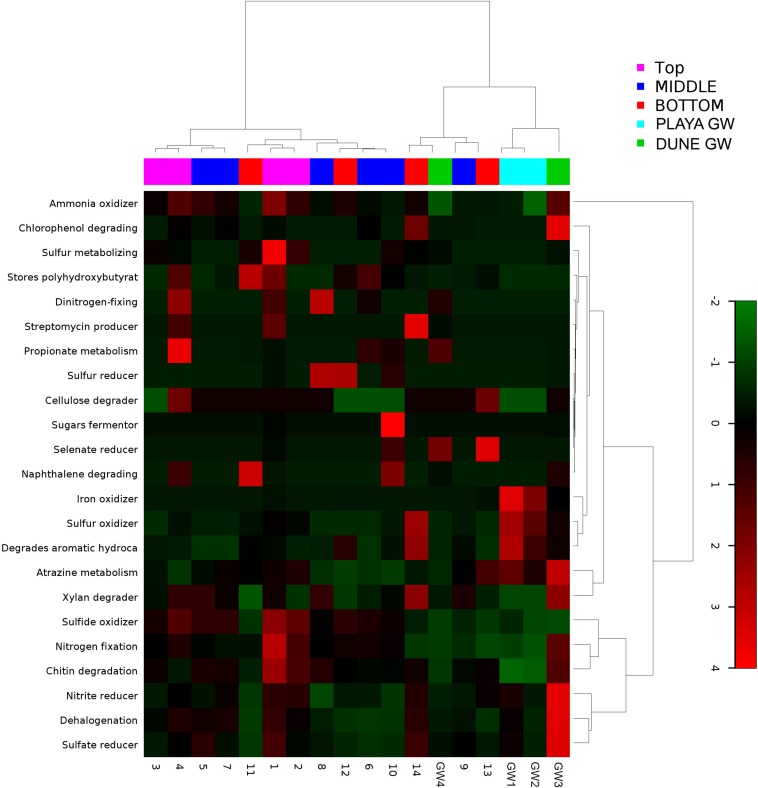
Heatmap of 23 bacterial metabolic potentials constructed from 16S rRNA-based taxonomic identities. The color scale represents relative intensity of each metabolic pathway, with red being the highest and the green the lowest.

**FIGURE 10:**
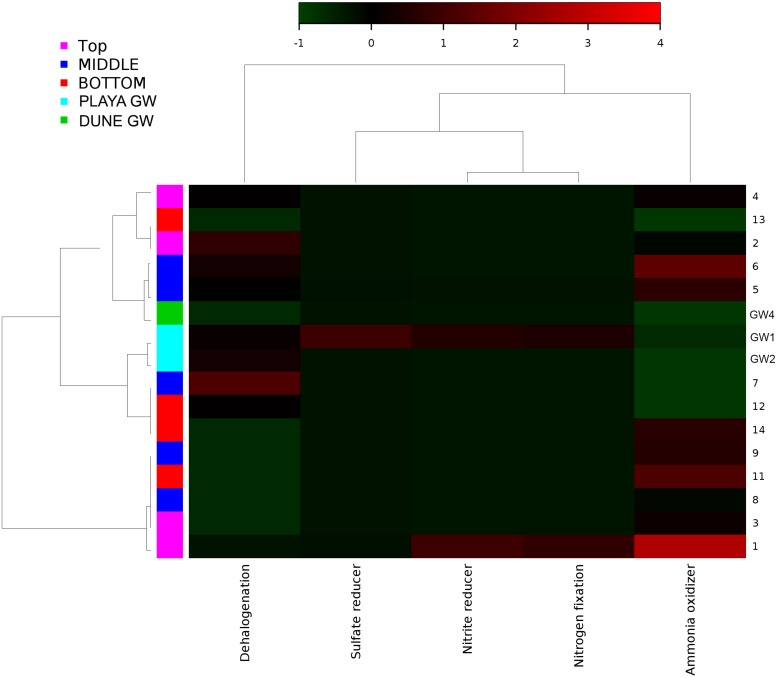
Heatmap of archaeal metabolic potentials constructed from 16S rRNA-based taxonomic identities. The color scale represents relative intensity of each metabolic pathway, with red being the highest and the green the lowest.

**FIGURE 11:**
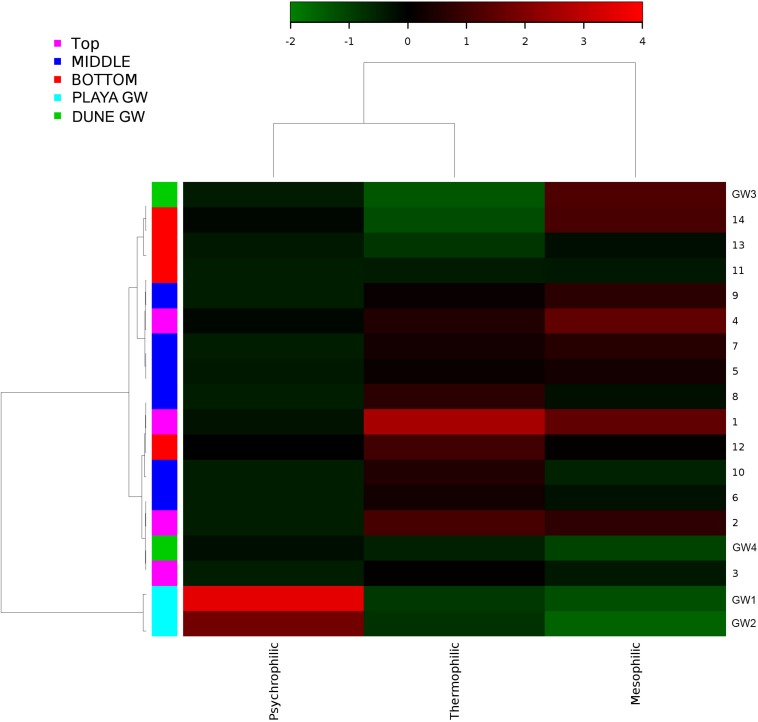
Heatmap of bacterial temperature adaptation inferences constructed from 16S rRNA-based taxonomic identities. The color scale represents relative intensity of each metabolic pathway, with red being the highest and the green the lowest.

Metabolic inferences of prokaryotic communities further indicate that microorganisms had colonized the subsurface sediments and groundwater taking advantage of diverse micro-environmental conditions (Figures 9–11).

## Discussion

Desert microbial communities strategically inhabit near-surface environments where they have the availability of sunlight and evaporation triggered chemical disequilibria; at the same time, they remain protected from the desiccation and UV radiation by a thin layer of sediment (e.g., Madigan et al., 2015). Our results show that overall near-surface samples contain more bacterial species that the bottom samples (Table 3). However, the diversity indices suggest that in general, the microbial diversity is relatively low and that diversity varies, and it seems to be increased at 50 cm (sample 4) and 90 cm (sample 9) depths. This suggested that near-surface samples contained diverse species, but with few dominate species that are well-adapted to the extreme conditions in this environment. Furthermore, this study shows that there are layers of sediments, possibly with similar geochemistry or similar physical conditions (comprised of two or more samples: Samples 1 and 2; Samples 4 and 5) that poses similar microbial communities (Figures 9–11). This observation is in accord with results of Sirisena et al. (2018), where they showed that similar microbial communities are present in sediments with similar geochemistry characterized by depth profiles at Lake Lucero ecosystem. The taxonomic, metabolic, and temperature inferences indicate that aerobic organisms are likely the dominant constituent of the microbial communities, although anaerobes and microaerophiles are also present throughout the sediment column and groundwater samples and that organisms have adapted to different temperature regimes that exist in groundwater (presence of psychrophiles) and sediment column (thermophiles and mesophiles). Based on the predominance of ${\text{NO}}_{3}^{-}$ over ${\text{NH}}_{4}^{+}$ and the taxonomic data analyzed it appears that the environment is oxygenated throughout the depth column. Overall, the organisms observed here are generally consistent with those found in other playas and hypersaline environments (Mesbah et al., 2007; Costa et al., 2008; Oren, 2008; Navarro et al., 2009; Makhdoumi-Kakhki et al., 2012; Babavalian et al., 2013).

**TABLE 3:** Diversity metrics from bacterial and archaeal 16S rRNA gene Illumina Miseq sequencing.

**Sample**	**No. of reads**	**No. of OTUs**	**Diversity indices**		**Evenness indices**		**Estimated richness**		**Coverage**
						
			**Simpson**	**Shannon**	**Simpson**	**Shannon**	**Chao 1**	**ACE**	**Good**
**Bacteria^a^**									
1	132,470	1,515	19.061178	4.718459	0.07429	0.747621	743.732058	967.250947	0.993249
2	79,517	812	21.988785	3.738441	0.250134	0.780696	397.041124	799.701548	0.994271
3	99,017	782	19.956364	3.619609	0.232723	0.759208	389.72082	896.242879	0.994169
4	117,298	899	36.639509	4.198392	0.349154	0.842351	398.779234	595.293092	0.99463
5	69,815	897	23.519454	3.980187	0.245591	0.804175	406.277957	806.39352	0.993948
6	66,297	770	23.111976	3.786453	0.220272	0.779215	388.356512	1239.246434	0.993596
7	105,162	706	16.867009	3.541933	0.214934	0.76288	308.724164	676.405993	0.994505
8	99,196	666	18.440478	3.5071	0.239623	0.768525	294.563452	609.696937	0.993955
9	118,886	697	19.739544	3.462572	0.240779	0.747145	332.211666	871.631594	0.994041
10	109,096	765	21.695474	4.107581	0.166903	0.807015	299.639737	303.734827	0.993275
11	81,756	711	14.023681	3.32267	0.225944	0.737564	425.100165	960.777085	0.994471
12	96,591	972	22.88627	4.085189	0.240819	0.813293	381.884719	637.456159	0.99393
13	83,903	548	11.454561	3.044709	0.145956	0.671205	229.294937	440.621694	0.994713
14	85,670	479	15.926822	3.120422	0.243184	0.728504	257.006363	748.584071	0.995031
GW-1	110,430	933	8.370894	3.560572	0.095972	0.709815	373.632901	787.195774	0.993874
GW-2	49,781	746	11.784472	3.546838	0.066229	0.662226	315.290841	335.071847	0.992447
GW-3	146,284	1,409	5.417147	3.736143	0.055545	0.664866	565.168842	1000.074716	0.993102
GW-4	83,477	1,311	71.262153	5.389618	0.208565	0.873887	554.577184	507.720065	0.991274

**Archaea^b^**									

1	13,946	130	3.594778	1.760895	0.413368	0.709585	16.5885	34.675379	0.996917
2	10,694	48	2.762361	1.149301	0.469699	0.642542	6.435833	12.567437	0.997569
3	3,204	34	1.48348	0.175187	0.69195	0.54049	2.165	0.082	0.99407
4	5,244	31	2.945836	1.296066	0.676964	0.796717	5.676667	9.24569	0.996758
5	6,190	77	3.340255	1.52502	0.611924	0.774539	9.48275	22.716393	0.993215
6	9,213	50	3.07368	1.393258	0.642189	0.798194	6.08	7.495332	0.997069
7	10,204	33	2.962945	0.888007	0.695001	0.740935	3.9525	1.730232	0.998138
8	1,781	23	1.036928	0.139464	0.737398	0.592432	1.8585	0.019	0.991016
9	3,525	40	1.057984	0.218579	0.638017	0.481737	2.5715	0.081111	0.992908
11	17,291	58	2.064032	0.183981	0.67752	0.521257	2.2265	0.082	0.997687
12	2,658	29	1.037109	0.322761	0.513639	0.347212	3.342333	0.598285	0.992476
13	5,404	34	1.039981	2.416355	0.522688	0.816609	27.70005	56.4991	0.995929
14	3,674	22	1.019328	0.07938	0.843008	0.747722	1.464	0.006	0.996189
GW-1	11,395	120	6.497753	2.134756	0.699867	0.860418	15.07845	16.888131	0.994208
GW-2	50,788	299	6.001275	2.517272	0.474777	0.809518	29.270993	56.417967	0.996909
GW-4	8,385	68	8.062561	1.714997	0.536062	0.775391	10.50305	14.292014	0.995587

*^*a*^Samples were rarefied to the smallest sample size (49781 reads) for the diversity, evenness, richness, and coverage calculations. ^*b*^Samples were rarefied to the smallest sample size (1781 reads) for the diversity, evenness, richness, and coverage calculations. Samples 10 and GW-3 contain very low number of Archaeal reads (42 and 59 respectively). Therefore, these two samples were eliminated from subsequent analyses: diversity, cluster, and metabolic inference analysis.*

### Groundwater

The analysis of groundwater samples shows that the microbial communities at WSNM differ greatly based on their playa or dune field location (Figure 8). Furthermore, groundwater samples varied significantly even within the same site (e.g., the two dune groundwater samples had low similarity) indicating a highly contained nature of these communities. The microbial communities of the dune groundwater samples are largely differentiated from each other by the presence of *Pseudomonas*, *Sphingobium*, and the class *Rhodobacteraceae*, which are significantly more abundant in the deep aquifer (GW-4). *Pseudomonas* specific OTUs are the only group with a significant presence in both samples (>10,000 sequences). *Sphingobium* and *Tepidimonas* are genera of obligate aerobes, so their high abundances may indicate that both aquifers are aerobic (Moreira et al., 2000; Chen et al., 2013). *Seohaeicola* contains aerobic and anaerobic species, as well as moderate halophiles (Yoon et al., 2009; Xie et al., 2014). All of the genera noted here are gram-negative. Archaeal data are not available for the shallow aquifer (GW-3); however, the deep aquifer contains more *Thaumarchaeota* and *Woesarcheota* than playa groundwater or the sediment; which may be indicative of ammonia oxidation at this location. The dune groundwater revealed the predominant presence of eukaryotic clade *Viridiplantae*, which are photosynthetic green algae adapted to live in extreme environments of desert soil (e.g., Lewis and Lewis, 2005) to deep marine water environments (Zechman et al., 2010).

Similar to the dune groundwater, in general, the playa groundwater samples differ from each other but are more similar to each other than to dune groundwater samples (see Figure 8). The groundwater below the sediment column (GW-1) has a higher abundance of the genera *Halomonas*, *Marinobacter*, and *Thiomicrospira* while the groundwater in southern Lake Lucero (GW-2) is more abundant in *Sediminimonas*, *Halobacteria*, *Methanomicrobia*, and the uncultured E6ACO2 (*Bacteroidetes*). The only OTU abundant at both locations denotes an unclassified *Gammaproteobacteria*. Similarly to the dune groundwater, gram-negative and aerobic microbes are prominent. Halophile (*Halobacteria*) have a more substantial presence in GW-2 (southern edge of the playa), implying higher salinity at this location (Jeong et al., 2013; Madigan et al., 2015; Zhong et al., 2015), which is confirmed by our conductivity measurements where GW-1 measured 59 μS and GW-2 139.9 μS (Table 1). The abundances of *Thiomicrospira* and *Sediminimonas* imply that both locations are primarily oxidizing environments with readily available reduced compounds (Wang et al., 2009; Madigan et al., 2015). Based on metabolic inferences, playa groundwater prokaryotes have a high potential for aerobic metabolic pathways (oxidize iron and sulfur, fix nitrogen to degrade various organic compounds), however, some organisms with sulfate and nitrate reducing metabolic potentials are available too (Figures 9, 10). Bacterial temperature adaptation inferences indicate that groundwater community is adapted to daily and seasonal extreme temperature changes as organisms with the highest intensity indices belong to psychrophiles (Figure 11). Unclassified eukaryotic phyla dominate groundwater sample GW-1, while *Alveolata* and *Stramenopiles* are next relevant groups in GW-2 and GW-1. *Stramenopiles* are mostly represented as diatoms with flagella that would facilitate life in groundwater (Madigan et al., 2015).

A moderate number of methanogenic archaea are observed in the uncultured order STK1210A of the class *Methanomicrobia* (5,000–10,000 sequences) (Madigan et al., 2015). The vast majority of these are present in playa GW-2; methanogens have previously been reported in the groundwater of Lake Lucero (Schulze-Makuch, 2002) and subsurface sediments (Sirisena et al., 2018). Previous work has shown that hydrogenotrophic methanogens (such as the *Methanomicrobia*) tend to be inhibited in the presence of sulfate-reducing bacteria, as these microbes consume acetate and H_2_ needed by the methanogens (Smith et al., 2008; Garcia-Maldonado et al., 2015).

### Sediment and Groundwater Interactions

The mixing effect generated by the capillary action of a very shallow groundwater table makes the subsurface of Lake Lucero a very dynamic environment that intermittently receives moisture from the groundwater even when the surface gets very dry. This dynamism plays a significant role in structuring microbial communities. Although the composition of microbial communities identified within the sediments and groundwater differ significantly (Figure 7), it seems that the capillary action of the groundwater causes a limited redistribution of microbes throughout the column preventing microbial segregation by depth, in which some of the microbes appear to be displaced. For example, the presence of purple phototrophic bacteria deep in the sediment column, where they would have limited or no access to sunlight, they can survive under these conditions but cannot grow optimally (Casamayor et al., 2008). It is important to note that, in general, the playa’s groundwater microbial community is more similar to the sediment community than that of the dune groundwater, although the similarity percentage is low. The stable stratification seen in microbial mats in wetter settings cannot be maintained under these dry and occasionally moistened conditions, and thus the community composition in Lake Lucero seems to be different from that seen in environments with a salt crust and microbial build-ups. Similarly, Canfora et al. (2014) show that the presence or absence of such crust is a significant differentiating factor amongst the microbial communities of saline environments.

Similarly, to more stable wet settings, the dry environments that maintain somewhat continuous environmental conditions will exhibit microbial stratification too (Sirisena et al., 2018). Sirisena et al. (2018) have observed microbial stratification at the edge of the Lake Lucero. The difference in this and their sampling points is that the lake edge dries sooner than the center (topographic low) and the groundwater table is deeper at the edge (they were not able to reach the groundwater table during the sampling), so the sediment was dry throughout the sampled profile. The lack of groundwater allowed for the development of anoxic communities on the bottom of the sampling site, and this is not the case in this central part of the playa where the oxidizing groundwater and associated capillary action are influencing community structure and environmental chemistry. Surprisingly groundwater and sediment contain communities with dramatically different adaptations to temperature regime. The groundwater may get easily cooled close to freezing during the winter time when the night temperatures get bellow the freezing point, and the organisms living in the water consist of psychrophiles (Figure 11). The sediments may get hot during the day and the organisms living in the sediments are mostly represented by thermophilic and mesophilic forms. Additionally, we had hypothesized that the longer presence of readily available water at the center of the lake may influence the increase in diversity when compared to other, drier, parts of the playa (such as the location described at the edge of the lake in Sirisena et al., 2018). When comparing the diversity indices among the data, Simpson indices are showing overall higher values for the central location, and Shannon indices are about the same; which may be indicative of some increase in diversity at the center of the lake vs. the edge of the lake.

### Distribution of Organisms Along the Sediment Profile

Although the capillary action of groundwater causes mixing of microbial organisms, we have observed some trends too. The deepest part of the sediment column contains sticky clays, a slight increase in ${\text{NH}}_{4}^{+}$ concentrations, lower ${\text{NO}}_{3}^{-}$ concentrations, and the presence of saprophytes. However, ${\text{NO}}_{3}^{-}$ levels are still higher than ${\text{NH}}_{4}^{+}$ and saprophytes are low in abundance, and samples 12 to 14 metabolic potential (Figure 9) is the highest for ${\text{NH}}_{4}^{+}$ and sulfide oxidation, and Chitin degradation indicating that even at this depth, the playa sediments may still remain aerobic.

*Proteobacteria* were present in all samples and became more abundant with the depth. They were also more prevalent in groundwater, except the deep dune groundwater (GW-3); in the shallow dune groundwater, this phylum composed more than 80% of the bacterial OTUs. Such high presence of *Proteobacteria* may be due to the gram-negative nature of the *Proteobacteria*; their cell walls would make them less susceptible to osmotic lysis caused by sudden influxes of water during the wet season (Madigan et al., 2015). The *Gammaproteobacteria* genus *Pseudomonas* has a large presence throughout all samples, especially in the sediment (Madigan et al., 2015). SIMPER analysis showed that OTUs identified as *Pseudomonas* were significant differentiating factors for sample 14 and the dune groundwater samples. However, without species-level identification of the OTUs and more in-depth analysis (e.g., mRNA analysis), it is difficult to understand this distribution. Conversely, *Acidobacteria* and *Gemmatimonadetes* were abundant in most sediment samples but mostly absent from groundwater samples.

*Bacteroidetes* are significantly more abundant in the upper half of the sediment column than the bottom part, possibly due to the increased availability of cellulose and chitin closer to the surface (Figure 5). *Bacteroidetes* are typically saccharolytic (specializing in the degradation of complex polysaccharides such as cellulose and chitin) (Madigan et al., 2015), and polysaccharides could be available at WSNM from the plants and arthropods that have been seen on and near the surface (Stroud, 1950; Muldavin et al., 2000). The *Actinobacteria* are similarly less abundant in deeper sediment samples, and even less abundant in groundwater samples. The most significant of these are the family *Acidimicrobiales*, in particular, the OM1 clade; the single largest OTU in the dataset belongs to this class (approximately 102,000 sequences). They are more abundant in samples 1–13 of the sediment column than in sample 14 or the groundwater, as was shown via SIMPER analysis. This uncultured group of *Actinobacteria* has been observed often in freshwater and marine environments and is thought to be oligotrophic and planktonic (Morris et al., 2012; Ghai et al., 2013; Mizuno et al., 2015). In contrary, the *Firmicutes* are generally more abundant in deeper sediment samples and have a marginal presence in the groundwater samples.

Another example of localized distribution within community structure is bacterial phylum *Chloroflexi*, which is mostly present in samples 11–13 and the groundwater beneath the sediment column. Their distribution in the sediment correlates with the increase in the concentrations of Fe, K, Mg, and Ti ions in samples 11–13, the change in lithology (presence of sticky dark brown to black clay), and direct exposure to groundwater capillary action. This correlation may indicate that groundwater capillary action may provide higher availability of nutrients at this depth. Aside from the already mentioned green non-sulfur bacteria, the most abundant classes of the *Chloroflexi* are uncultured groups (JG30-KF-CM66 and S085) and unclassified OTUs. The unknown OTUs and JG30-KF-CM66 drive the *Chloroflexi* abundance in samples 11 to 13.

In comparison to groundwater, the sediment samples are more consistent in their microbial communities. The most distinct is sample 14, which is a relative outlier from the rest of the sediment column due to negligible presence of *Acidimicrobiales* OM1 in sample 14 as opposed to its high abundance in the rest of the sediment. These planktonic bacteria flourish during the wet season and lay dormant during the dry season, in our case they are the most abundant at the surface and seem to be redistributed throughout the sediment column most likely via capillary action (Morris et al., 2012; Ghai et al., 2013; Mizuno et al., 2015). Additionally, sample 14 contains a much higher abundance of *Acinetobacter*, a diverse bacterial genus with most free-living soil species that are saprophytes (Doughari et al., 2011). Only *Pseudomonas* is identified as being consistently abundant throughout the sediment column. The archaeal communities were less consistent than the bacterial, the sample 12 was isolated, and samples 7 and 13 were paired while the rest of the samples grouped in a dendrogram (Figure 7). Samples 7, 12, and 13 all had a negligible abundance of *Thermoplasmatales*, while the other samples had them in high abundance. The samples 7 and 13 also had a uniquely higher abundance of *Thaumarchaeota* (ammonia oxidizers), while 12 was unique for its high abundance of the halophilic genus *Halapricum* (Campbell et al., 2011; Song et al., 2014). Although the clustering of the *Halapricum* population in sample 12 is curious, it implies that the communities in this sample are not too different from 7 to 13 since halophiles are ubiquitous in all samples and *Halapricum* is not significantly different from other *Halobacteria* genera; additionally, 7 has a high abundance of unclassified *Halobacteria* (Song et al., 2014). Therefore, it seems that the main difference between the archaeal communities of samples 7, 12, and 13, and the rest of the sediment is the abundance of *Thermoplasmatales*. Considering that the metabolic inferences do not reveal any taxonomic grouping based on metabolic potential (Figure 10), it seems that differences within the archaeal communities derive from the fact that most of the Archaeal OTUs were not classified up to Genus level when compared to bacterial OTUs. Further, the upper levels of the sediment (samples 3, 4, and 5) show the presence of fungi and *Viridiplantae*, which may be organismal remnants of the ephemeral lake that withdrew their presence to the last near surface hydrated habitats. Fungi are mostly represented by the subphyla *Pezizomycotina* (phylum *Ascomycota*), a highly diverse group that includes saprophytes, plants, and mutualists (Kumar et al., 2012).

### Halophiles and Oligotrophs

The hypersalinity is one of the most dominant characteristics of Lake Lucero environment, and halophiles have a substantial presence in the examined population. The identified archaeal OTUs are from the classes *Halobacteria* and *Methanomicobia* (unclassified halophile ST-12K10A), and the phylum *Nanohaloarchaeota*; which are common for hypersaline environments (Sorenson et al., 2005; Falb et al., 2008; Oren et al., 2009; Barton and Northup, 2011; Garcia-Maldonado et al., 2015; Madigan et al., 2015; Di Meglio et al., 2016). The uncultured groups of order *Thermoplasmatales* are identified with a significant presence in the sediments; some of these uncultured groups have been previously reported in the saline environments (Siam et al., 2012; Madigan et al., 2015).

Halophiles are present in all the dominant bacterial phyla: *Proteobacteria, Actinobacteria, Firmicutes, Chloroflexi*, and *Bacteroidetes* (Madigan et al., 2015). The purple sulfur bacteria order *Chromatiales* (*Proteobacteria*) contains some of the most extreme bacterial halophiles and has been observed in similar hypersaline environments (Sorenson et al., 2005). A highly abundant (>10,000 sequences) halophile is the nitrite and nitrate-reducing genus *Sediminimonas* in the groundwater of southern Lake Lucero (Wang et al., 2009). Halophiles observed in moderate abundance (5,000–10,000 sequences) include the genera *Salinibacter*, *Staphylococcus*, *Streptococcus*, and *Nitriliruptor* (Mesbah et al., 2007; Anton et al., 2008; Makhdoumi-Kakhki et al., 2012; Madigan et al., 2015). *Nitriliruptor* is alkaliphilic (Sorokin et al., 2009). Low abundance (1,000–5,000 sequences) halophiles include the genera *Rothia*, *Kocuria*, and *Truepera* (Albuquerque et al., 2005; Chou et al., 2008; Tang et al., 2009).

The halophiles observed are diverse, and they include extreme (*Chromatiales)*, moderate (e.g., *Nitriliruptor*) and slightly (e.g., *Truepera*) halophilic groups (Albuquerque et al., 2005; Sorokin et al., 2009). The distribution of halophiles is relatively uniform, which implies that there is no significant salinity gradient within the sediment column. This observation is consistent with relatively uniform concentrations of Na in these samples. Additionally, the detected halophiles are predominantly aerobic (Costa et al., 2008; Navarro et al., 2009; Makhdoumi-Kakhki et al., 2012; Babavalian et al., 2013; Canfora et al., 2014).

The genus *Ralstonia* is observed in abundance, at the sediment surface (samples 1 and 2), the groundwater and the sediment interface (samples GW-1 and14), this genus contains oligotrophic organisms (such as *R. pickettii*) that are common in water and soil (Ryan et al., 2007). Another oligotrophic phylum, *Gemmatimonadetes* is highly abundant (Fawaz, 2013). These organisms are commonly observed in arid soils and are well-adapted to living in low moisture conditions, but they are not known to be well-adapted to wet-dry cycles of playa environment; therefore in here, they are present in lower abundance at the surface and very low abundance in the groundwater (Fawaz, 2013). The previously mentioned *Acidibacteriales* OM1 is also oligotrophic (Mizuno et al., 2015). The deficiency in available nutrients in Lake Lucero makes this a natural habitat for oligotrophs, so their abundance and wide distribution in this environment are anticipated (Madigan et al., 2015).

### Photosynthetic Organisms

A moderate amount of green non-sulfur bacteria (anoxygenic phototrophs found in a wide range of environments) and purple sulfur bacteria were observed but the phylum *Chlorobi*, which consists of green sulfur bacteria, is present in very low abundance (Madigan et al., 2015). *Cyanobacteria* are observed in very low abundance. It is possible that the mixing effect of the groundwater capillary action limits the growth of phototrophs since it prevents the segregation of microbial communities, which would allow for stabilization in the most favorable position. This mixing would also have the potential to redistribute phototrophs to deeper levels of the sediment where they could be living in a state of dormancy (Jones and Lennon, 2010).

### Nitrogen Cycle

Portions of the nitrogen cycle at Lake Lucero were assessed through the concentrations of ${\text{NH}}_{4}^{+}$, ${\text{NO}}_{3}^{-}$, and the OTU abundance data. The ${\text{NO}}_{3}^{-}$ concentrations are 5.97 to 22.94 times higher than the ${\text{NH}}_{4}^{+}$ in analyzed samples, indicating the oxidizing environment and nitrogen oxidation as a possibly important microbial process. Organisms that possess capabilities to contribute to nitrogen cycling have been identified in the samples (Figures 9, 10). However, based on our data, we cannot say with certainty whether they used these capacities and in what measure. Metabolic potential map indicates that bacterial and archaeal organisms have potential for nitrogen and dinitrogen fixation, ammonia oxidation and nitrate reduction (Figure 9), making them capable of utilizing and cycling available ${\text{NH}}_{4}^{+}$ and ${\text{NO}}_{3}^{-}$ compounds. A moderate amount of green phototrophic bacteria capable of nitrogen fixation are observed in samples 11–13 (Wahlund and Madigan, 1993). A small amount of nitrogen-fixing purple phototrophic bacteria are observed, predominantly at the surface (sample 1). Additionally, they were found in samples 8, 11, 12, and the deep aquifer (GW-3) sampled at the dune field (Wahlund and Madigan, 1993). No other known nitrogen-fixing microbes are explicitly identified. These coincides with the mapped nitrogen fixing pathways (Figures 9, 10) in prokaryotes from samples 1, 2, 4, 8, and 12 as well as GW-3. Nitrate reduction has been inferred in top most samples 1 and 2 and in groundwater samples GW-1 and 3 and for sample 14, at the bottom of the sampled interval. A very small amount of anaerobic ammonium oxidizing (anammox) bacteria of the order *Brocardiales* are observed, only in the deep aquifer of the dune groundwater (Jetten et al., 2009). Rhizobial genera are identified in large amounts (mostly *Bradyrhizobium*) and are well-distributed throughout the sediment column but with comparatively low abundance in groundwater (Madigan et al., 2015). The absence of observed plants here indicates that rhizobia likely live freely in the soil, in which state they cannot fix nitrogen (Madigan et al., 2015).

The distribution of green and purple bacteria in the sediment profile implies that nitrogen fixation is possible at the surface; additionally, the input of nitrogen into the system is likely augmented by atmospheric deposition too (Fenn et al., 2003). In our samples, the distribution of potential ammonia-oxidizers generally mirrors that of the potential nitrogen fixers. The small number of nitrifying microbes is distributed throughout the sediment column. These observations, as well as a large difference in ${\text{NH}}_{4}^{+}$ and ${\text{NO}}_{3}^{-}$ concentrations, indicate that the environment along the depth profile is aerobic and that nitrification processes could be important in this system. The dune groundwater seems to have a predominant presence of organisms capable of nitrification, although the brine-based groundwater is differentiated by the presence of anammox bacteria. Both, ${\text{NO}}_{3}^{-}$ and ${\text{NH}}_{4}^{+}$ concentrations are generally low, as is typical in arid environments (Madigan et al., 2015). The slight increase in ${\text{NH}}_{4}^{+}$ concentrations at the bottom of the sediment profile implies the possible presence of the decaying organics or denitrification and DRNA (dissimilative reduction of nitrate to ammonium) that may occur here. Further, a moderate abundance of *Petrimonas* in sample 11 (contains the species *P. sulfuriphila* which reduces elemental sulfur and nitrate and is a mesophilic anaerobe) correlates with the darker coloration of sediments at this depth, which additionally suggests the presence of decaying organic matter (Grabowski et al., 2005). *Coryneform* bacteria are aerobic saprophytes that are also present in low abundance at this depth; by degrading organic matter, they release ammonium into the soil (Madigan et al., 2015). However, even in this part of the sediment column, ${\text{NO}}_{3}^{-}$ concentrations are still 7.03 times higher than ${\text{NH}}_{4}^{+}$ concentration.

### Sulfur Cycle

All of the WSNM environments are sulfur dominated, so it is interesting to inquire whether these resources are available and potentially used by microbial organisms inhabiting the area. From our work, we are only aware of the mineral substrates rich in sulfur element, as gasses or water composition had not been analyzed. The purple sulfur bacteria may use H_2_S as an electron donor (or elemental sulfur if H_2_S is limited) and often rely on sulfate-reducing and/or sulfur-reducing microbes to produce H_2_S (Madigan et al., 2015). Their occurrence in samples 11 and 12 coincides with the presence of the sulfur-reducing *Petrimonas* detected in sample 11 (Grabowski et al., 2005). Despite the abundance of gypsum and other sulfur-bearing minerals in this environment, only a small number of sulfate-reducers and sulfur-reducers are observed, although the highly abundant genus *Pseudomonas* contains species that are capable of sulfur-reduction (such as *P. mendocina*) (Madigan et al., 2015). Inferred sulfur reducing metabolic pathways were observed in samples 8, 12, 10, while sulfate reducers were observed in samples 1, 5, 14 and in dune groundwater (GW-3). The low abundance of sulfate-reducing microbes is likely due to the predominantly aerobic environmental setting, and the samples are not rich in organic matter to use as electron donors (Madigan et al., 2015). We have not observed any living plants near our sampling site; no biofilm or decaying plants were detected in any of our samples by eye or using SEM/EDS. Possible carbon sources would include chitin from the exoskeletons of arthropods, and the phototrophic microbes observed in the sediment (Madigan et al., 2015).

*Thiomicrospira* is the only sulfur-oxidizing genus observed, but it is highly abundant and mostly distributed in sample GW-1 (at the bottom of the sampled sediment profile), with a minor presence at the surface (Sorokin et al., 2006a, b; Madigan et al., 2015). This coincides with the metabolic inferences heatmap where GW-1, GW-2, and sample 14 were mapped with the highest intensity for sulfur oxidation. The relative lack of sulfur oxidizers in the sediment column implies that most of the sediment has a low amount of reduced sulfur compounds compared to the groundwater, as would be likely in a primarily aerobic setting with a large number of sulfate minerals. The presence of *Chromatiales* and *Thiomicrospira* at the surface suggests that there is a source of reduced sulfur in this environment. The minor presence of purple sulfur bacteria in the dune groundwater implies the same at that location. Nonetheless, it seems that oxidative processes would dominate the sulfur cycle in Lake Lucero sediments, which would be consistent with the assessment of the nitrogen cycle and the microbial populations. Overall, the organisms with the metabolic potential of sulfur cycling are not abundant in this environment even though sulfur dominates the chemistry of mineral substrate, which seems not to be bioavailable.

## Conclusion

The sediment and groundwater of Lake Lucero are highly dynamic environments, strongly characterized by the extreme seasonality of the climate, the capillary action of the groundwater, and the hypersalinity. This extreme environment harbors microbial communities that are primarily aerobic, gram-negative, and are largely characterized by their survival adaptations.

Halophiles and oligotrophs are extremely common throughout all samples, as anticipated. The observed communities are very diverse and contain organisms capable of different metabolic pathways: methanogens, phototrophs, heterotrophs, saprophytes, ammonia-oxidizers, sulfur-oxidizers, sulfate-reducers, iron-reducers, nitrifiers, and denitrifiers.

The microbial diversity varied significantly between groundwater and sediment samples. The dynamism of this environment manifests in the relatively consistent character of the sediment hosted microbial communities, where significant distinctions are more taxonomic than phenotypic. Taxonomically, the dune and playa groundwater organisms will group separately from each other and separately from sediment. Temperature adaptation inferences revealed that organisms living in the groundwater are likely psychrophiles, while organisms inhabiting the sediment column consist of thermophiles and mesophiles that can withstand elevated temperature regime. Additional ecological partition is observed as a change in the deepest part of the column as the communities are affected by the presence of sticky clays, higher concentrations of various cations, and potentially decaying organic material. Saprophytes and *Chloroflexi* are more abundant in these lithologies. The salinity appears to be lower, as indicated by the decrease in Na concentration, and a lower abundance of extreme halophiles relative to the rest of the sediment profile.

Complex ecosystems as such as the one of Lake Lucero may have existed on Mars in the past and we have demonstrated in this paper and in Sirisena et al. (2018), that subsurface of desert environment may host very diverse organisms and diverse environments. We hope that the variety of the discussed environments may inform future astrobiological explorations.

## Data Availability Statement

Raw Illumina sequencing data was archived in SRA (Sequence Read Archive) at NCBI under the BioProject number: PRJNA558907.

## Author Contributions

All authors contributed to the intellectual input and assistance on this project, and approved the final version of the manuscript. SR, MG, and IW collected the data. SR, KS, and MG contributed to the data processing and analysis. MG collected the samples. SR and MG wrote the manuscript.

## Conflict of Interest

The authors declare that the research was conducted in the absence of any commercial or financial relationships that could be construed as a potential conflict of interest.
